# Can Multi‐Vertebral CT‐Based Finite Element Models Accurately Predict Strains? An In Vitro Validation Study

**DOI:** 10.1002/cnm.70085

**Published:** 2025-08-10

**Authors:** Alessandra Aldieri, Chiara Garavelli, Luca Patruno, Marco Palanca, Marco Viceconti

**Affiliations:** ^1^ Polito^BIO^MedLab, Department of Mechanical and Aerospace Engineering Politecnico di Torino Torino Italy; ^2^ Department of Industrial Engineering Alma Mater Studiorum ‐ University of Bologna Bologna Italy; ^3^ DICAM, Alma Mater Studiorum ‐ University of Bologna Bologna Italy

**Keywords:** digital image correlation, FE model validation, IVD constitutive modelling, multi‐vertebrae FE model, vertebral fracture prediction

## Abstract

Many proposed FE models to predict the vertebral risk of fracture consider single vertebrae only, neglecting the role of the intervertebral discs in load transmission and distribution across vertebrae. Inclusion of the intervertebral discs in multi‐vertebrae models would allow more physiological boundary conditions. However, while CT allows material properties to be assigned to the vertebrae, no information about the discs is provided. Hence, the aim of this study was to build multi‐level FE models uniquely based on CT data and validate them by comparing the predicted displacements and strains against the experimental measurements. One spine segment (T10‐L1) was harvested from a human spine and tested in flexion‐compression in the elastic regime. During the test, displacements and strains on the anterior surface were measured with digital image correlation. The FE model was built starting from the CT scan of that same spine segment. HU‐based isotropic linear elastic properties were assigned to the vertebral bone. Five different combinations of hyperelastic material properties from the literature were assigned to the discs, modeling the nucleus pulposus and the anulus fibrosus separately. The boundary conditions replicated the flexion‐compression test performed experimentally. Predicted displacements and strains on the vertebrae surfaces were compared against the measured displacements and strains. The model excellently predicted the displacement field (*R* ^2^ = 0.92/0.99). On the other hand, different constitutive laws for the discs resulted in different principal strain distributions, which substantially differed from the experimental one, showing average relative errors higher than 34%. In conclusion, a different modeling approach should be adopted for the discs in CT‐based multi‐level FE models to achieve acceptable accuracy.

## Graphical abstract

The study presents the development of a subject‐specific CT‐based multi‐level FE model of the spine and the comparison of its predictions with in vitro experimental data. CT images, which require assumptions about the intervertebral disc to be made, might not be sufficient to allow the development of an accurate FE model.

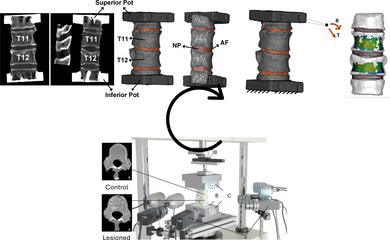

## 1 Introduction

Low‐energy vertebral fractures represent a very severe event, which implies increased morbidity, mortality, and decreased quality of life in already fragile subjects [1], in addition to economic implications for the healthcare systems [2]. On the one hand, vertebral fractures are related to aging due to risk factors such as increased propensity to fall and deteriorated mechanical properties of the bone tissue [3]. On the other hand, an increasing incidence of bone metastases has been observed, primarily at the spine [4], compromising the vertebrae's physiological mechanical competence [5, 6]. Accurately predicting fracture risk would be pivotal in implementing appropriate preventive interventions and effective treatment strategies. The currently adopted clinical gold standards for bone fracture prediction have shown limited accuracy in stratifying subjects at high risk of fracture from subjects who are not. T‐score, for example, suffers from poor specificity and sensitivity in predicting hip and vertebral fractures connected to osteoporosis [7]. Similarly, the Spinal Instability Neoplastic Score (SINS) suffers from a certain degree of subjectivity in the final clinical decision [8] and lacks sufficiently good specificity [9].

In the last decades, several Computed Tomography (CT)‐based digital twin approaches have unravelled the possibility of adopting *in silico* tools to provide information about bone resistance non‐invasively [10, 11, 12, 13]. *In silico* methodologies have successfully outperformed the gold standard [14, 15, 16] for some anatomical sites, such as the femur. On the contrary, for several reasons, the vertebral fracture prediction *in silico* is not as straightforward. The vertebrae, indeed, are interposed with the intervertebral discs (IVDs) which play a crucial role in load transmission and distribution. Still, modelling accurately the IVDs opens significant challenges, including structural complexity, patient‐specific variability, and the need for appropriate material model selection [17, 18]. This is the main reason why the vertebral fracture risk is often predicted by only modelling one vertebra [19, 20, 21] or neglecting the IVDs and endplates [22, 23]. However, this approach intrinsically poses some limitations since it does not allow the application of physiological loading conditions on the vertebral bodies [24, 25]; it does not consider to what extent IVD degeneration might impact load transmission to the adjacent vertebrae [26]; it neglects a failure location [6] and the different failure modes [27].

Including the IVD in the model allows accounting for a more physiological loading condition of the spine. In principle, this would require the inclusion of a comprehensive description of the IVD through magnetic resonance imaging (MRI) as an additional input to the model besides CT [28, 29, 30]. Nevertheless, developing a digital twin for vertebral fracture risk prediction based only on the subject's CT would be much more clinically feasible; although no information would be provided regarding the disc, forcing us to model it with population material properties coming from the literature.

In this light, the aim of this work was to assess the accuracy of a multi‐vertebrae FE model uniquely based on CT, where the IVDs were included and modeled according to constitutive models and parameters found in the literature.

## 2 Materials and Methods

A patient‐specific FE model of a spine segment with evidence of metastatic disease was developed from CT images of a cadaveric sample. A total of five constitutive models for the nucleus pulposus (NP) and the annulus fibrosus (AF) were adopted for modeling the IVDs mechanical response. The vertebral strains obtained from the model were compared to experimental data acquired through Digital Image Correlation (DIC) [31] on the same sample, and the accuracy of the proposed modeling strategy was assessed.

### 2.1 Mechanical Testing

**FIGURE 1:**
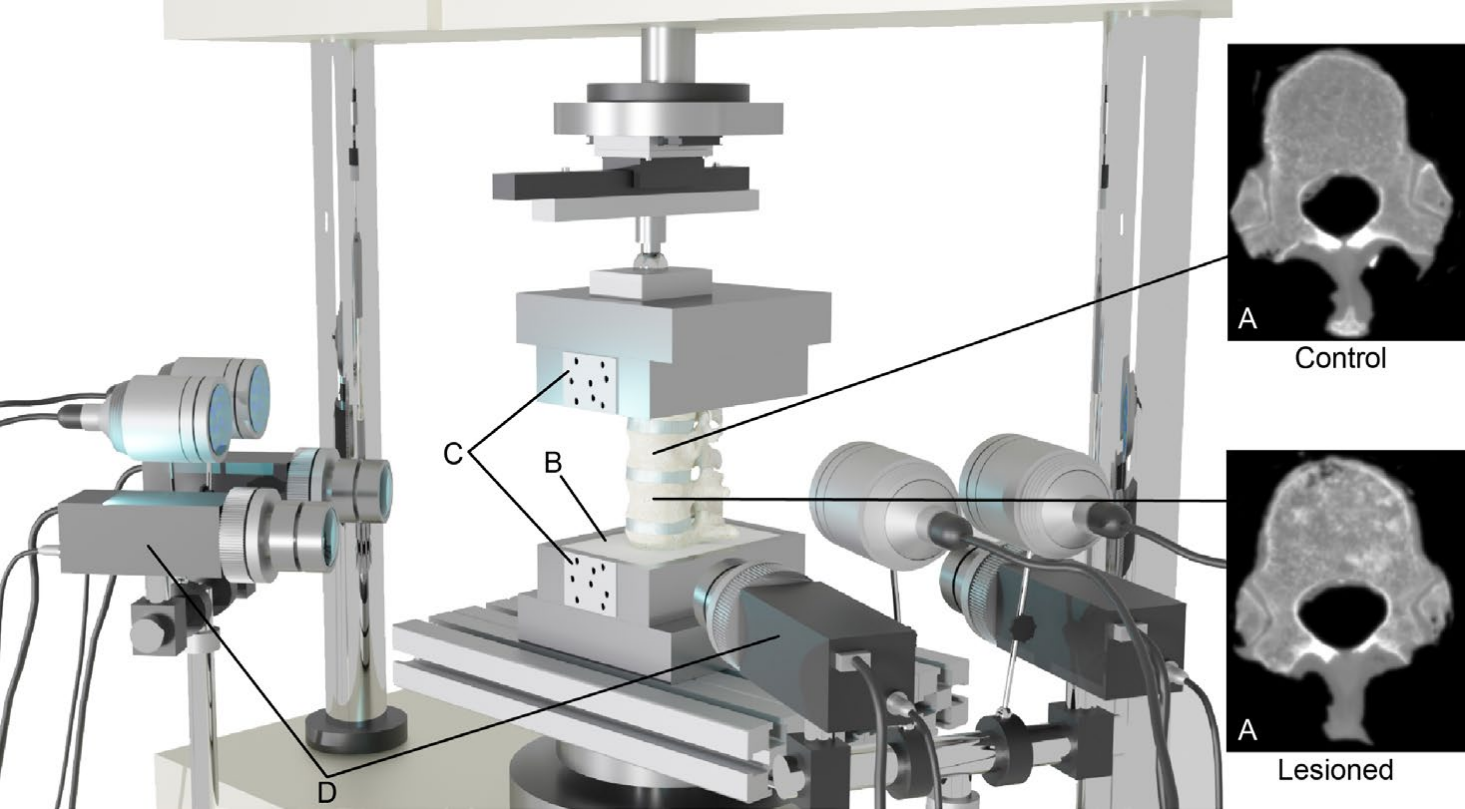
Experimental test set‐up. The specimen with one control (A, top) and one metastatic (A, bottom) vertebra is placed into the testing machine (B). Pots displacements are tracked thanks to glued markers (C). The flexion‐compression test is recorded by a three‐dimensional DIC system (D).

Experimental tests were performed on a thoracolumbar cadaveric specimen obtained from an ethically approved donation program (Anatomic Gift Foundation Inc.). The sample included four vertebrae and the interposed three IVDs, from T10 to L1. T11 was considered the healthy control of the specimen, while T12 showed signs of lytic metastatic lesions (Figure 1A). Half of the most cranial and half of the most caudal vertebrae were embedded in polymethyl‐methacrylate (PMMA) cement (Figure 1B). The anterior longitudinal ligament was removed in order to expose the vertebral bodies and the intervertebral discs surfaces. After the preparation of the specimen, diagnostic images were acquired with a spiral CT (AquilionOne, Toshiba, Japan), to be subsequently used for the FE model development. Scans were performed at 120 kVp with 200 mA tube current, 0.24 × 0.24 mm pixel size, and 1 mm slice thickness. Afterwards, the specimen was sprayed with white water‐based paint (Q250201 Bianco Opaco, Chreon, Italy) diluted at 40% with water [32, 33] (without any background) to generate a unique speckle pattern recognizable by a 4‐cameras 3D‐DIC system (Aramis Adjustable 12 M, GOM, Braunschweig, Germany, with 12mPixels cameras and 75 mm metrology‐standard lenses) [32, 33]. Flat circular markers were also glued on the aluminum pots of the test machine in order to track the displacement of the superior and inferior pots through the DIC system (Figure 1C). Blue LED lights were used to illuminate the specimens and the markers.

The mechanical flexion‐compression tests were performed using a uniaxial testing machine (Instron 8500 controller with Instron 25 kN load cell, Instron, UK) in a displacement‐controlled modality (Figure S1 in the Supporting Information). The load application point was shifted forward by 10% of the anteroposterior dimension of the central IVD to impose an anterior flexion [34]. A ball joint and low friction bearings allowed the top pot to rotate and translate freely, while the bottom was totally constrained. The load magnitude was identified as the load able to induce a physiological minimum principal strain range [35] (i.e., in the order of 2500/3000 microstrain) on the anterior surface of the T11 control vertebra. The experimental reaction force was recorded. During the test, DIC images of T11 and T12 and the two pots were acquired at 25 Hz (Figure 1D). DIC measured the displacements and derived the strains on the visible vertebral surfaces. Systematic and random errors were identified in a zero‐strain configuration, where the specimen was kept unloaded and thus where any displacement or strain different from zero was accounted for as an error. Systematic and random errors settled at around 10 and 25 μm for displacement and 30 and 100 με for strain, respectively. Processing of the DIC images was performed using a facet size of 30 pixels, a grid spacing of 10 pixels, a median spatial filter on 5 facets, and a median temporal filter on 2 frames. Considering a pixel size of around 0.3 mm, these parameters allowed us to obtain a measurement spatial resolution of about 2 mm.

Further details regarding the mechanical testing procedure are extensively described in [36].

### 2.2 Finite Element Analysis

The FE model corresponding to the thoracolumbar cadaveric specimen tested previously was constructed starting from the available CT scans. The steps required to develop a CT‐based homogenised FE model have been presented in detail in our previous work [37] and will be briefly summarised here. Firstly, CT images of the specimen were segmented to extract the geometry of the vertebral bodies, IVDs, and pots separately. A threshold method based on Hounsfield Units (HU) values was used to pick out bone tissue and PMMA cement (200/3000 HU for bone and 1100/1800 HU for PMMA), followed by manual editing (Mimics 25.0, Materialise NV, Leuven, Belgium). The IVDs profiles were approximated through a simplified geometry, with adjacent vertebrae endplates used as upper and lower bounds. Posterior processes were removed considering that the posterior ligaments would activate in flexion only if the physiological range was exceeded (at this spinal level, the neutral zone and the range of motion for each FSU are around 0.6° ± 0.1° and 3.5° ± 0.8° respectively [38]), which did not occur in the here presented experiments (total flexion: 2.8°).

**FIGURE 2:**
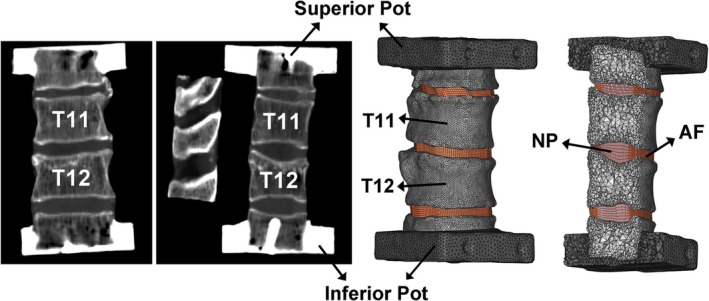
Anterior and mediolateral views of the specimen's CT scan on the left. On the right is the reconstructed FE model. Labels identifying T11, T12 vertebrae and the PMMA pots in both CT and model, as well as nucleus pulposus (NP) and anulus fibrosus (AF) in the models, are included.

A 10‐node tetrahedral structural solid mesh was generated for the vertebrae and PMMA pots. In contrast, a 20‐node hexahedral structural solid mesh was built for the IVDs (Hypermesh v2019, Altair Engineering Inc.). The maximum edge length was imposed equal to 1 mm following a sensitivity analysis. Figure 2 shows the CT scan employed to develop the FE models and the whole FE model.

Subject‐specific heterogeneous linear elastic isotropic material properties were assigned to the vertebral bone. In particular, a density‐elasticity relationship [39, 40] was adopted to map Young's modulus E (MPa) values element‐wise (Bonemat V3.1, Istituto Ortopedico Rizzoli, Bologna, Italy) [41] based on volumetric bone mineral density values $\rho_{\mathrm{app}}$ (g/cm^3^) derived from the CT HU values according to the following equations:

(1) $$\rho_{\mathrm{ash}}=0.079+0.877\cdot \rho_{\mathrm{QCT}}$$

(2) $$\rho_{\mathrm{app}}=\frac{\rho_{\mathrm{ash}}}{0.6}$$

(3) $$E=4730\cdot {\rho_{\mathrm{app}}}^{1.56}$$

$\rho_{\mathrm{QCT}}$ values were estimated from HU values using a linear relationship identified through a phantom‐based calibration procedure, where the European Spine Phantom (ESP) was scanned and a linear regression performed between the average HU values of its components and their known density values [37]. A Poisson's coefficient (*ν*) equal to 0.3 was assigned to all bone elements.

As no information about IVDs subject‐specific material properties could be derived from the available CT scans, a set of different constitutive laws was identified in the literature and adopted for the IVDs. More specifically, different material properties were assigned to the NP and AF, which were arbitrarily defined by imposing that NP occupied around 40% of the overall IVD volume, according to [42] (Figure S2 in the Supporting Information). Five different combinations of material models for NP and AF were employed to describe the mechanical behavior of the AF and NP, leading to five FE models that differed in how the IVDs were modeled only. Gasser‐Ogden‐Holzapfel (GOH) anisotropic hyperplastic constitutive law described the AF in all cases, although with different material parameters [43, 44]. NP was modeled using an isotropic hyperelastic Mooney‐Rivlin model [45, 46, 47] or an incompressible linear elastic model. Table 1 shows the five combinations of the constitutive laws adopted for AF and NP. Tables S2 and S3 in the Supporting Information report the specific parameters of the constitutive models employed for the AF and NP, respectively.

**TABLE 1:** Overview of the five different combinations of hyperelastic constitutive models adopted for NP and AF. When the same constitutive models have been employed with different parameters, a number has been added after the constitutive law's name.

	AF	NP	
Model 1 (M1)	GOH‐1	Mooney‐Rivlin‐1	[48]
Model 2 (M2)	GOH‐1	Incompressible elastic	[48, 49]
Model 3 (M3)	GOH‐2	Incompressible elastic	[49]
Model 4 (M4)	GOH‐3	Mooney‐Rivlin‐2	[50]
Model 5 (M5)	GOH‐4	Mooney‐Rivlin‐3	[50]

Linear elastic material properties were assigned to the PMMA pots, with *ν* = 0.3 and an elastic modulus equal to 3 GPa (obtained from in‐house experimental tests on cement samples).

**FIGURE 3:**
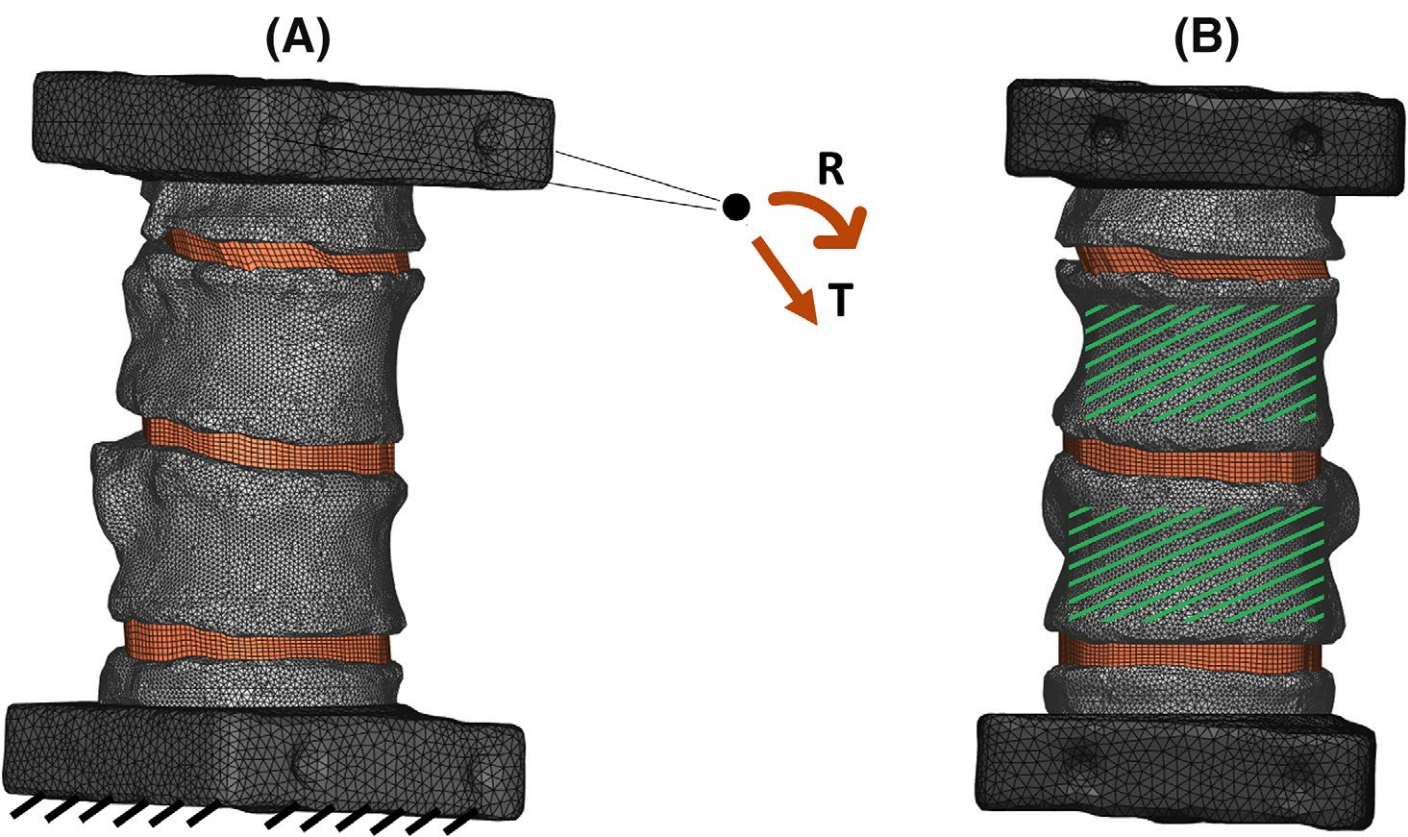
(A) The experimental boundary conditions replicated on the FE model: While the inferior pot was fully constrained, the superior pot was kinematically constrained to the displacement (rigid translation T and rotation R of a point identified as the centroid of the flat circular markers glued on the aluminium pots). (B) The FE model with highlighted in green the vertebral surfaces where the numerical and experimental outcomes were analysed. Most proximal and distal portions for each vertebra were not considered.

Boundary conditions were assigned to the FE model to replicate the experimental test conditions (Figure 3A). The metal and PMMA cement pots were assumed to be rigidly connected, so only the second ones were modeled. The positions of the superior metal pot at the initial and final steps of the experimental test acquired through the DIC were processed using a single value decomposition (SVD) algorithm to extract the rotation components R and translation T of the rigid pot's motions (Figure 3A). The obtained values were imposed on all the external nodes of the FE model superior PMMA pot using a multi‐point constraint. The inferior PMMA pot was fixed.

Static simulations were solved using Abaqus software (Dassault Systemes Simulia Corp., United States, v2021). Aiming to compare numerical outcomes with the available experimental information on T11 and T12 surfaces, the nodal displacements on the corresponding regions of the model (Figure 3B) were extracted; maximum and minimum principal strains were calculated by derivation, as described later in the next paragraph.

### 2.3 Comparison Between Experimental and Numerical Data

In order that a point‐wise comparison between experimental and numerical outcomes could be attainable, a first realignment step between the DIC and FE reference systems was carried out by adopting a procedure based on surface registration (Mimics 25.0) and SVD (Matlab v2020, MathWorks, Natick, Massachusetts, US). Specifically, a feature‐based rigid registration algorithm (Mimics, Materialise NV) was adopted to align the experimental DIC data of the two free vertebral surfaces in the initial (unloaded) configuration to the segmentation obtained from the CT data. Afterwards, the rigid transformation matrix (Mt) moving the original to the registered DIC point clouds was extracted using a SVD algorithm (Matlab v2020, MathWorks, Natick, Massachusetts, US), so that the coordinates and displacement vector components of all DIC points were transformed to the FE model reference system using Mt. The accuracy of the registration was assessed through the computation of a surface registration error as the root mean square error (RMSE) between the transformed DIC points and the nearest corresponding nodes on the model surface [37]. Subsequently, the DIC displacements on the vertebral surfaces were interpolated onto the locations of the vertebrae superficial nodes of the FE model. The interpolation was based on the inverse distance weighting algorithm employing the Euclidean norm with power equal to 2 and threshold radius set to 1 mm, accounting for the previously obtained surface registration error.

A point‐wise comparison between the numerical and the interpolated experimental displacement was performed to assess whether the models correctly reproduced the kinematics observed in the experiments and how different constitutive models for the IVDs impacted the kinematics. The linear regression determination coefficient (*R* ^2^) and root mean squared error normalized by the average measured value (%RMSE) (Equation 4) were computed, comparing DIC and FE local displacements.

(4) $$\%\text{RMSE}=100\cdot \frac{\sqrt{\frac{\sum {\left(x_{\mathrm{FE}}-x_{\mathrm{DIC}}\right)}^{2}}{N}}}{{\bar{x}}_{\mathrm{DIC}}}$$

with *N* being the number of points, $x_{\mathrm{FE}}$, $x_{\mathrm{DIC}}$ the FE and DIC variables and ${\bar{x}}_{\mathrm{DIC}}$ the average measured value.

Furthermore, superficial experimental and numerical strains on the vertebrae were obtained through derivation of the displacements under the hypothesis of a two‐dimensional deformation state. More in detail, maximum and minimum principal strains were considered and computed through derivation of the FE and DIC displacements on the triangular elements on the vertebral surfaces aiming to derive strains consistently between DIC and the FE models. Relative errors $\left(100\frac{\left|\varepsilon_{\mathrm{num}}-\varepsilon_{\exp}\right|}{\varepsilon_{\exp}}\right)$ and root mean squared error normalised by the average measured value (%RMSE) computed as shown in Equation (4) were the adopted metrics to compare numerical and experimental strain outcomes.

## 3 Results

**FIGURE 4:**
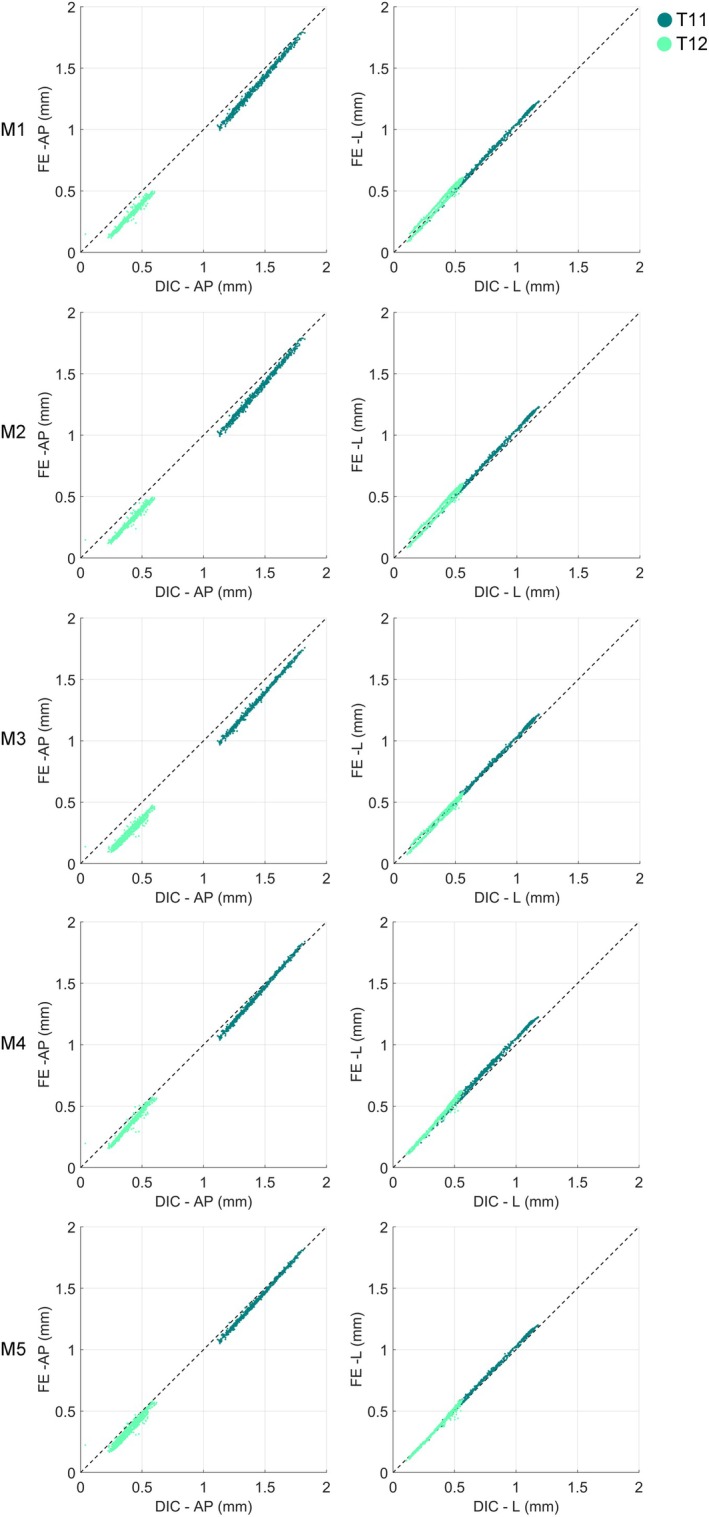
Comparison between computational and experimental displacements in the anteroposterior (AP) and longitudinal (L) directions computed on T11 (dark green) and T12 (light green) for the five different implemented models.

In Figure 4, the displacements on the T11 (healthy) and T12 (metastatic) vertebrae surfaces are shown; the DIC and FE outcomes for the five constitutive modeling laws employed to model the IVDs are compared. Displacements only in longitudinal (L) and anteroposterior (AP) directions were considered, disregarding displacements in the mediolateral (ML) directions due to the law of experimental accuracy along that specific direction.

An excellent agreement between the experiment and models is always achieved, only slightly affected by the IVDs' constitutive modeling adopted. Table 2 confirms these results, reporting linear regression determination coefficient (*R* ^2^) and normalized root mean squared errors (%RMSE).

**TABLE 2:** *R* ^2^ and normalised RMSE between the obtained computational and experimental displacements for the five models.

	*R* ^2^		RMSE%	
	T11	T12	T11	T12
M1	0.99	0.92	2.6	9.7
M2	0.99	0.92	2.7	10.1
M3	0.99	0.93	4.9	17.4
M4	0.99	0.96	1.1	5.2
M5	0.99	0.95	1.3	8.3

**FIGURE 5:**
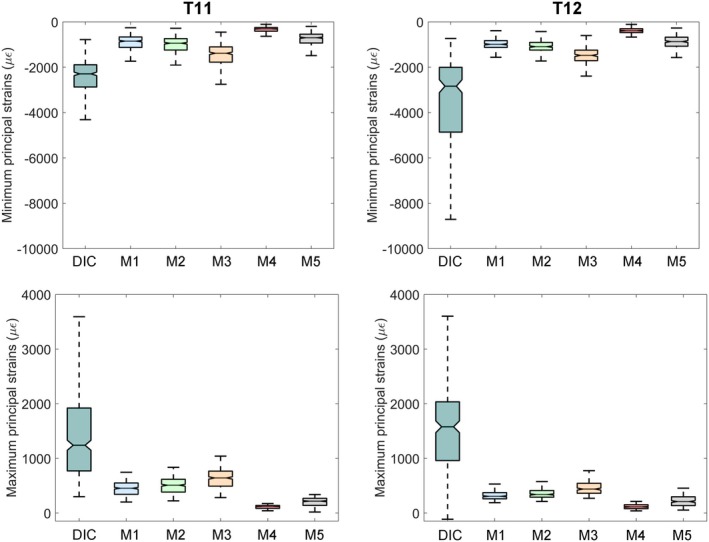
Boxplots comparing the principal strains' distributions on T11 and T12 between experimental DIC strains and those from the FE models. Notches allow for comparing the medians at the 5% significance level.

When deriving the displacements into principal strains, the different constitutive modelling affected the superficial vertebral strains to a larger extent compared to displacements, although none of the implemented material models for the IVDs allowed to achieve a numerical strain distribution comparable to the experimental one (Wilcoxon signed rank test *p* < 0.05), neither for the minimum, not the maximum principal strains, as clearly visible in Figure 5.

**FIGURE 6:**
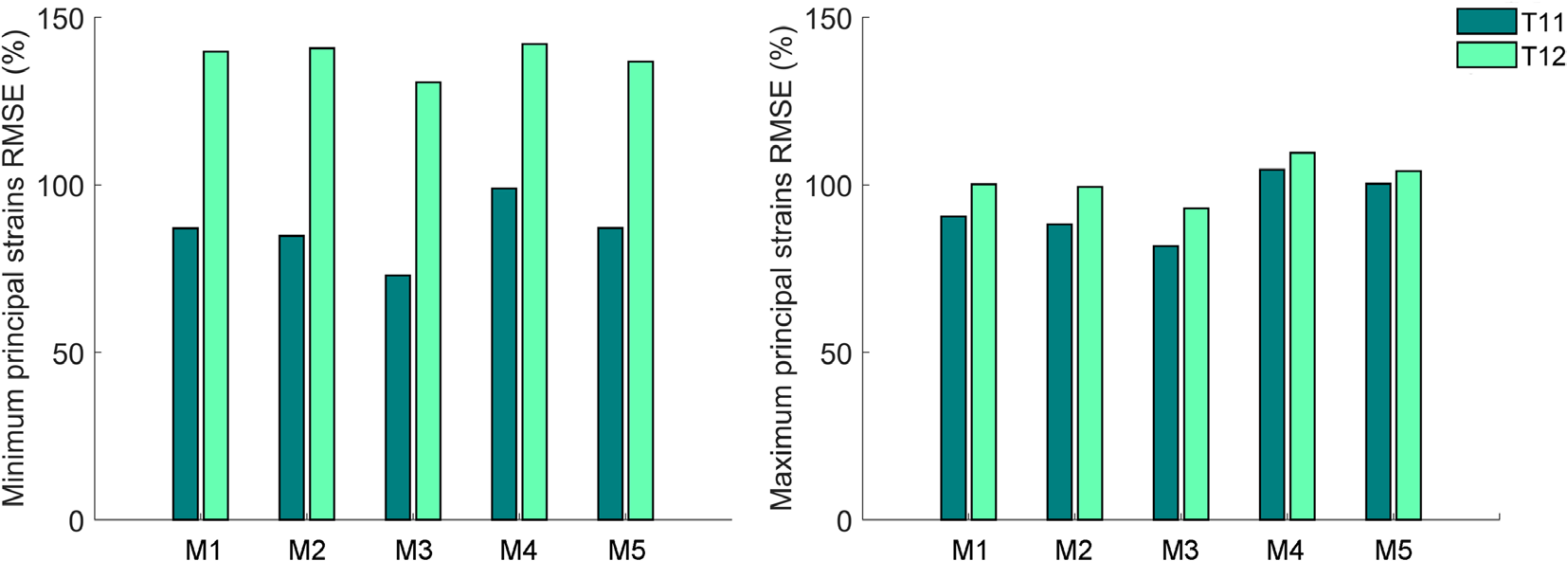
Normalized RMSE values computed comparing T11 and T12 principal strains between models and the experiment.

Figure 6 displays the normalized RMSE for the five models. As visible, considerable error values are obtained, consistently higher on T12 than on T11.

**FIGURE 7:**
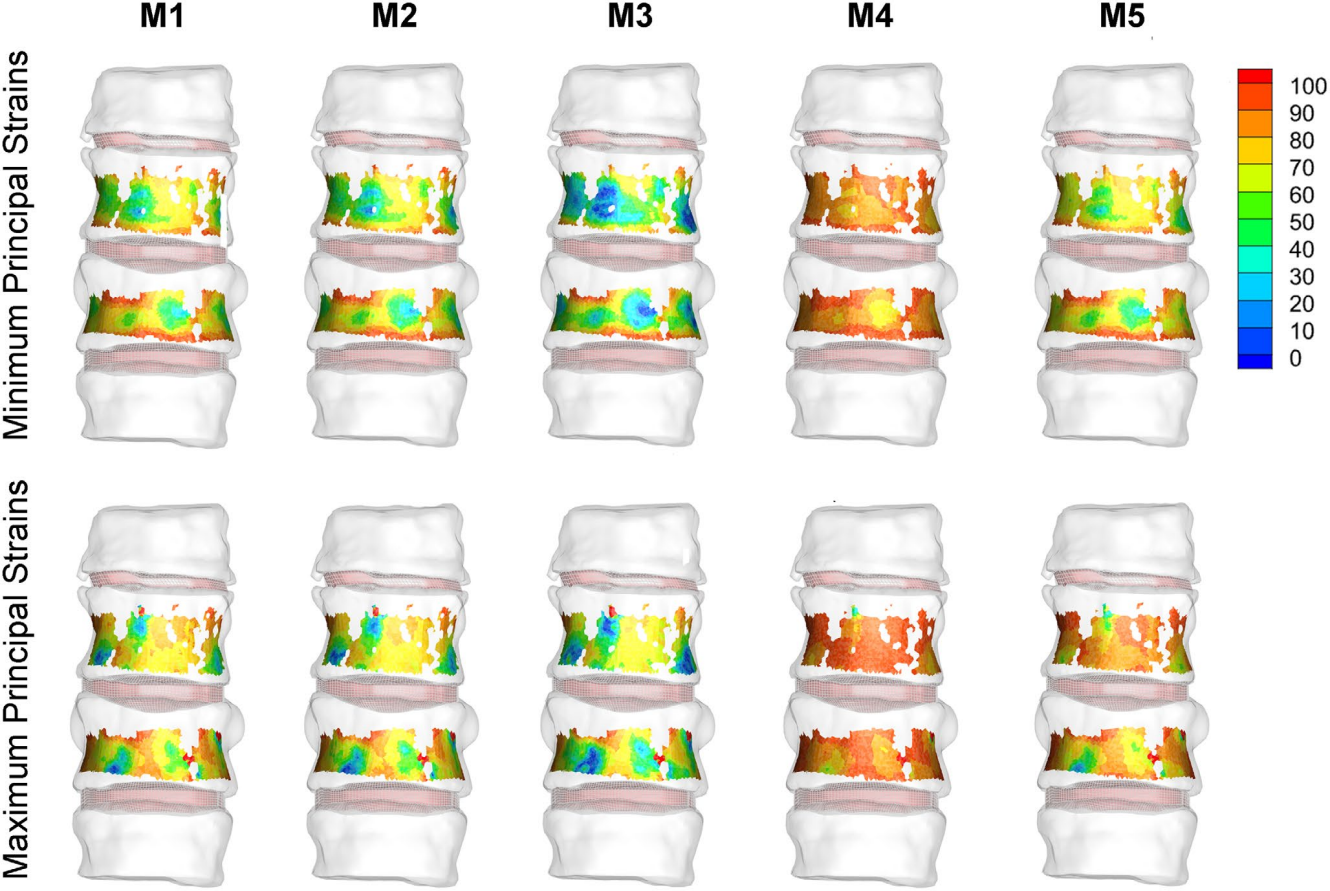
Contour plots showing the distributions of the relative error (%) on T11 and T12 surfaces for the five implemented models.

The best model was Model 3 (M3), which also exhibited, on average, the lowest relative error values for strains. In particular, M3 yielded 34% and 43% average errors on minimum and maximum principal strains, respectively, for the T11 (control) vertebra and 53% and 49% average errors on minimum and maximum principal strains for the T12 (metastatic) vertebra. In Figure 7, the relative error distributions are depicted for the five analyzed models.

## 4 Discussion

The purpose of the presented study was to assess whether a subject‐specific multi‐vertebrae FE model uniquely based on CT images could provide accurate results compared to in vitro data. If so, such a framework could be regarded as a promising tool to be adopted in the future to support and improve the currently adopted tools for vertebral fracture risk prediction. Aiming to develop an FE model of the human spine uniquely from CT information, assumptions regarding the IVDs material properties, impossible to be inferred from CT, have to be made. Therefore, in this study, different constitutive laws and parameters were retrieved from the literature and were used to model the IVDs mechanical behaviour. The resulting FE outcomes were compared to the corresponding DIC experimental results to assess the model's accuracy [36].

CT‐based FE models for risk of fracture assessment in vertebrae have usually been developed considering only one vertebra as an isolated body [51, 52]. However, this strongly prevents the application of physiological loading conditions such as those provided by the IVDs [53], which play a crucial role in load transmission among vertebrae [25], as well as the occurrence of high strain concentration close to the endplates, where fracture often originates [6]. Since IVDs can deeply influence the vertebrae's deformation state [54], *in silico* vertebral fracture prediction without IVD could be jeopardized, as the application of physiological loading conditions would be prevented. The crucial role played by the disc was highlighted in the work of Hussein and coworkers [55], where experimental compression tests were carried out, loading the vertebra through the IVDs and employing digital volume correlation (DVC), the volumetric extension of the DIC, to measure the displacements. That allowed the development of single vertebra FE models where the actual displacements detected on the superior vertebral endplate through DVC and a uniform compression were applied. Considerable differences were observed by comparing numerical and experimental vertebral displacements in the two cases (DVC‐derived BCs: *R* ^2^ = 0.66, percentage differences = 9%–48%; uniform idealised BCs: *R* ^2^ = 0.02, percentage differences = 20%–240%), highlighting how the development of reliable predictive models would require the inclusion of at least one functional spine unit.

On the other hand, accurate and patient‐specific modeling of the IVD mechanical behavior would require additional imaging information, for example, from MRI. In fact, MRI images would allow the anatomical distinction between AF and NP, thus enabling the IVD geometry personalization. Moreover, T2 relaxation times extracted from MRI images are related to the hydration state of soft tissues and could be related to personalized mechanical properties corresponding to specific degeneration stages [56, 57]. This clearly implies that CT alone would not be sufficient to build a subject‐specific multi‐vertebrae FE model for predicting the fracture risk at the spine, hampering its application in clinical practice. In this context, the main objective of the presented study was, therefore, to assess whether a CT‐based multi‐vertebrae FE model could be a sufficiently accurate predictor of vertebrae deformation state.

Our findings show how adopting different constitutive modeling strategies for the IVDs resulted in significant differences (*p* < 0.05) in the principal strains on the vertebral surfaces. Yet, although predicted displacements turned out to be in good agreement with those obtained experimentally, which was expected in light of the imposed displacement‐controlled boundary conditions, none of the implemented material models for the IVDs, coupled with the heterogeneous isotropic linear elastic modeling of the vertebral bone, could yield outcomes in terms of deformation in agreement with the experimental outcomes.

To the best of the authors' knowledge, only another study where full‐field validation on the vertebral surface strains field was performed [58] has been published, although it is based only on a single vertebra modeling. There, a good accuracy was found in the longitudinal strains prediction, with 50% of the nodes showing a 12.5% accuracy. In contrast, a lower agreement with experimental strains was found in the circumferential strains, with a median error of 39% and 10% of the nodes over 100% error. Herein, if the best case (M3) was considered, 32% and 53% median relative differences were obtained for minimum and maximum principal strains on the control vertebra, respectively. In comparison, 51% and 73% average differences for minimum and maximum principal strains were found on the metastatic vertebra. None of the nodes showed errors higher than 90% on the control vertebra T11, while 9% and 4% on the T12 vertebral surface showed relative errors higher than 90%. In the study of Imai et al. [59], where single vertebrae were modeled, and the validation was focused on the comparison between the predicted minimum principal strains and experimental outcomes from four rosette strain gauges applied on the vertebral surface, a much better agreement was found between numerical and computational values (slope = 0.93, intercept = 74 με, *R* ^2^ = 0.84). Also, Clouthier et al. [60] worked on spine segment FE models. Still, they validated the ultimate load values, which are not available here in light of the conservative design of the study.

Other studies also tried validating single vertebral FE models by comparing experimental failure locations versus simulated strain contour [61] or damage accumulation [22]. No consistency between the highest strain regions according to the predictions and the experiments could be found herein. On the other hand, the vertebra experiencing the highest strain degree was consistent with DIC results.

This study presents some limitations, which are described below. A first critical consideration involves the IVDs modeling and, in particular, (i) the simplified geometry of the IVDs, which neglected the external bulging the IVD exhibits, and (ii) the identification of the annulus fibrosus and nucleus pulposus, which was carried out a priori as no real information about their relative volume was available. Additionally, more sophisticated FE models would be required to explore the influence of posterior ligaments and facet joints, which were omitted in this work due to their limited role in the considered loading conditions. The second area of limitations concerns the boundary conditions assignment. It was assumed that no relative motion occurred between the cranial potted vertebra and the cement it was potted in and between the cement and the metal pot in which it was inserted and locked. However, this assumption could be judged reasonable due to the considerably greater stiffness of the metal pots compared to the specimen, also considering the secure connection ensured by the screws. The third point concerns the material properties assigned to the vertebral bone. First, isotropic linear elastic material properties were assigned to the vertebral bone, while the inclusion of anisotropic features might be more relevant for this mainly trabecular bone. In addition, the same constitutive laws were employed for all the vertebrae analyzed in the different studies without considering the vertebrae health status. While this choice may have introduced some inaccuracies, it was aligned with the findings of other authors who observed similarities in the mechanical properties of trabecular bone with and without lesions [23, 62]. This assumption holds when metastatic lesions can be characterized as low‐density bone tissue, as in the case of lytic lesions, while it might not be accurate enough for blastic lesions, and the specimen used for this study showed mixed metastases, with a limited occurrence of blastic lesions. Eventually, it should also be noted that the developed method was presented on one specimen only, considering the methodological nature of the work.

In summary, the obtained findings suggest that CT‐based subject‐specific multi‐vertebrae FE models, including HU‐based isotropic linear elastic material for the vertebral bone and the hyperelastic constitutive laws from the literature for the IVDs, would not be able to predict strain distribution on the vertebral surfaces accurately. Nevertheless, improvements could easily be implemented in the near future, such as the inclusion of a more realistic IVD's geometry and the adoption of different constitutive laws not only for the IVDs, but also for the bone, which could bring additional insights into the feasibility of CT‐based FE modeling of the spine for vertebral fracture prediction in silico. More specifically, the adoption of orthotropic instead of isotropic linear elastic properties might be more suitable for the vertebral bone, while as far as the IVD modeling is concerned, the implementation of more complex constitutive modeling strategies, such as multiphasic poroelastic modeling, might be worth investigating. Lastly, in light of the crucial role played by the IVD in load transmission among vertebrae, the possibility to estimate IVD subject‐specific material properties from clinical images, for example, MRI, would be pivotal in fostering the development of fully personalized multi‐level FE models of the spine able to achieve more accurate and realistic predictions.

## Conflicts of Interest

The authors declare no conflicts of interest.

## Supporting information


**Data S1:** Supporting Information.

## Data Availability

The data that support the findings of this study are available from the corresponding author upon reasonable request.
