# Supplementary material for: Can Multi‐Vertebral CT‐Based Finite Element Models Accurately Predict Strains? An In Vitro Validation Study
Source: Int J Numer Method Biomed Eng. 2025 Aug 10;41(8):e70085. doi: 10.1002/cnm.70085 (PMC12336364; doi:10.1002/cnm.70085)
Supplement: Supplementary file 1 — Data S1: Supporting Information. [file CNM-41-e70085-s001.docx]

**CAN MULTI-VERTEBRAL CT-BASED FINITE ELEMENT MODELS ACCURATELY PREDICT STRAINS? AN *IN VITRO* VALIDATION STUDY**

Alessandra Aldieri, Chiara Garavelli, Luca Patruno, Marco Palanca, Marco Viceconti

*Electronic Supplementary Material*

*
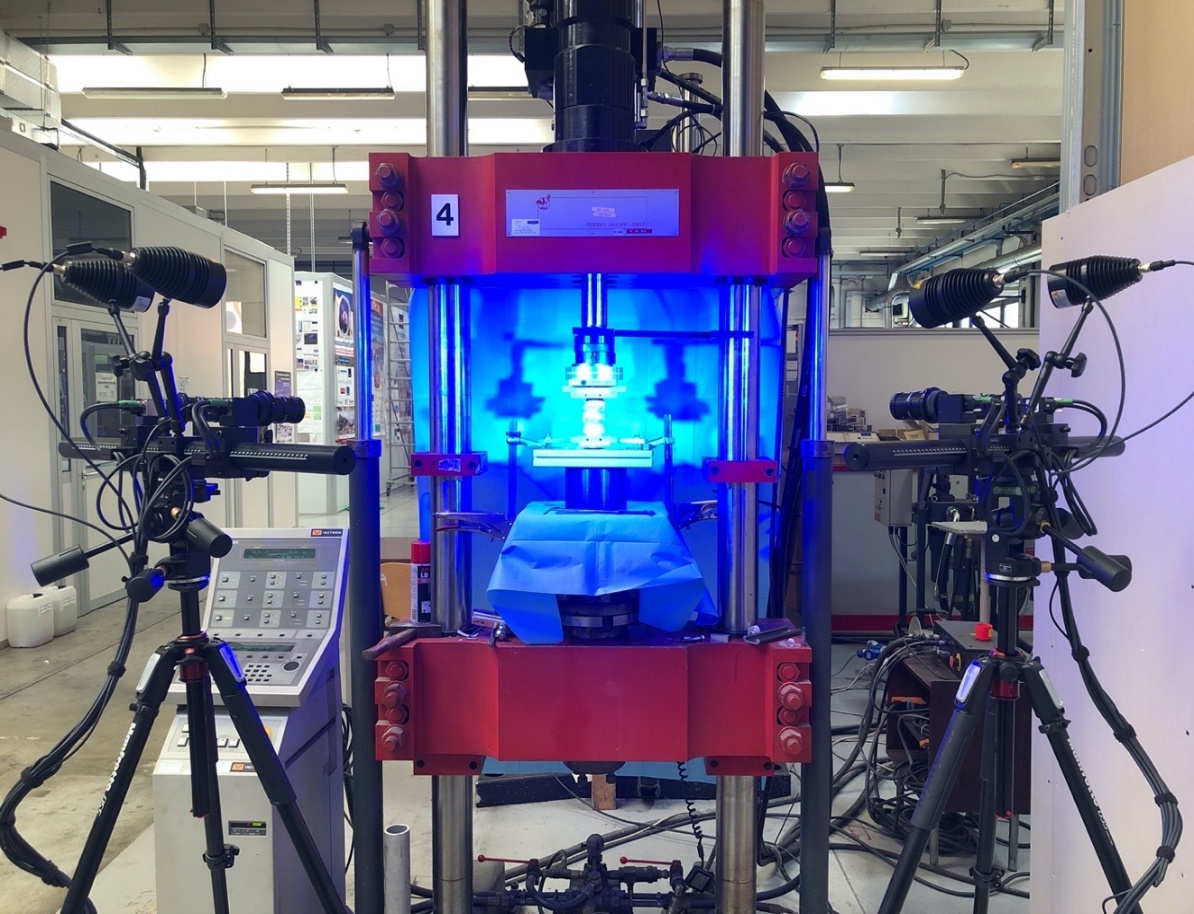
*

**Figure S1.** The real experimental setup. The testing machine with the specimen inside in visible in the centre, while the DIC cameras are visible at the two sides.


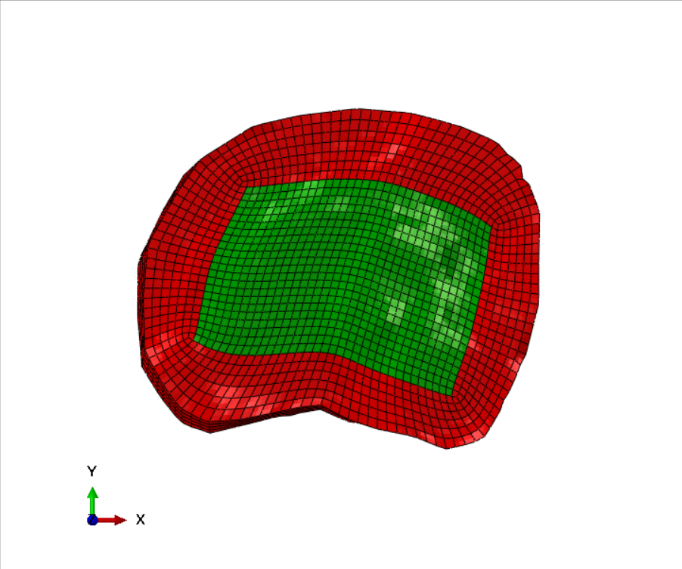

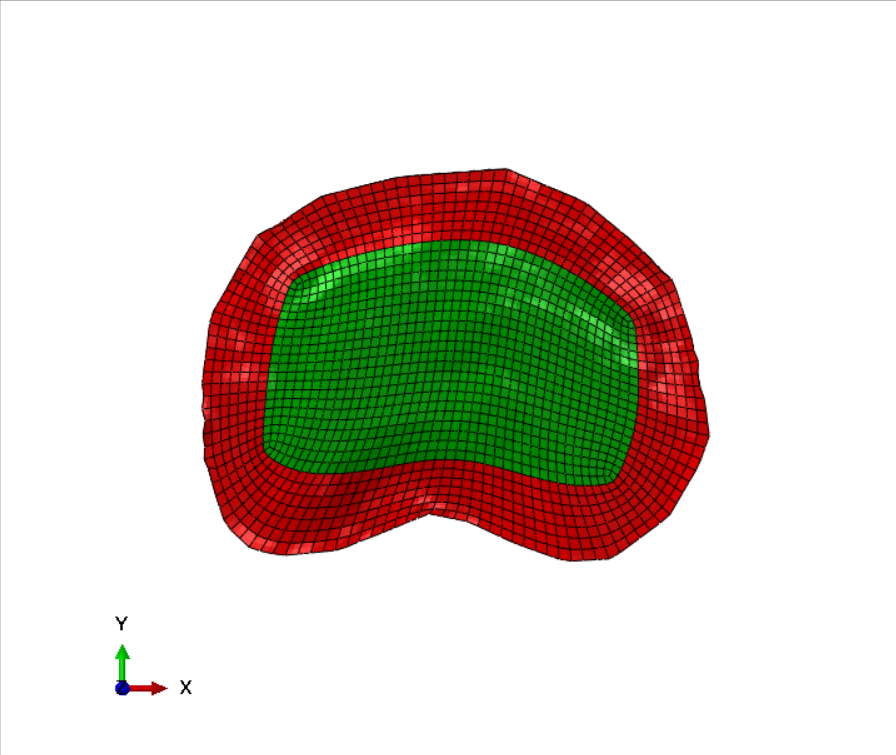

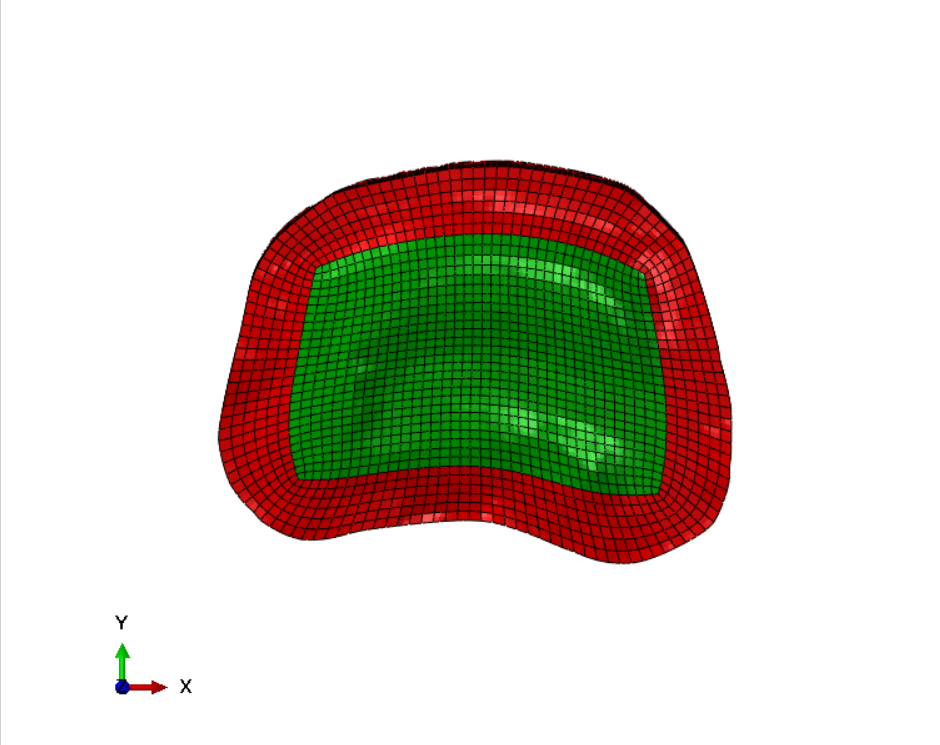


1. b) c)

**Figure S2.** Detail of the relative volume occupied by the anulus fibrosus (AF) and the nucleus pulposus (NP) on the three intervertebral discs included in the FE model: T10-T11 (a), T11-T12 (b), T12-L1 (c).

**Table S1.** Overview of the constitutive laws adopted for the anulus fibrosus (AF) and the nucleus pulposus (NP) in the five FE models created.

|  | **AF** | **NP** |  |
| --- | --- | --- | --- |
| **Model 1 (M1)** | GOH-1 | Mooney-Rivlin-1 | [36] |
| **Model 2 (M2)** | GOH-1 | Incompressible elastic | [36], [37] |
| **Model 3 (M3)** | GOH-2 | Incompressible elastic | [37] |
| **Model 4 (M4)** | GOH-3 | Mooney-Rivlin-2 | [38] |
| **Model 5 (M5)** | GOH-4 | Mooney-Rivlin-3 | [38] |

**Table S2.** Details of the anisotropic hyperelastic GOH law parameters employed to model the anulus fibrosus.

| **GOH-1** | $C_{10}=0.18 \left[ MPa \right], D_{1}=0.00667 \left[ MPa^{-1} \right]$  $Local direction=2$*,* $\kappa=0$  $External ventral:k_{1}=12 \left[ MPa \right], k_{2}=300, \vartheta=29^{\circ}$  $Internal ventral:k_{1}=1.74 \left[ MPa \right], k_{2}=43,5, \vartheta=29^{\circ}$  $External dorsal:k_{1}=3 \left[ MPa \right], k_{2}=60, \vartheta=41^{\circ}$  $Internal dorsal:k_{1}=0.435 \left[ MPa \right], k_{2}=8.7, \vartheta=41^{\circ}$ | [36] |
| --- | --- | --- |
| **GOH-2** | $C_{10}=0.4323 \left[ MPa \right] D_{1}=0 \left[ MPa^{-1} \right]$  $k_{1}=2.1638\left[ MPa \right],k_{2}=200.588$  $Local direction=2,$ $\vartheta=30^{\circ}$*,* $\kappa=0$ | [37] |
| **GOH-3** | $C_{10}=0.315 \left[ MPa \right]$, $D_{1}=0.254 [MPa^{-1}]$  $k_{1}=12\left[ MPa \right],k_{2}=300, \kappa=0.1$  $Local direction=2$ $\vartheta=35^{\circ}$*,* $\kappa=0$ | [38] |
| **GOH-4** | $C_{10}=1.13 \left[ MPa \right],D_{1}=0.14 [MPa^{-1}]$  $k_{1}=0.435\left[ MPa \right],k_{2}=8.7$  $Local direction=2,$ $\vartheta=35^{\circ}$*,* $\kappa=0.1$ | [38] |


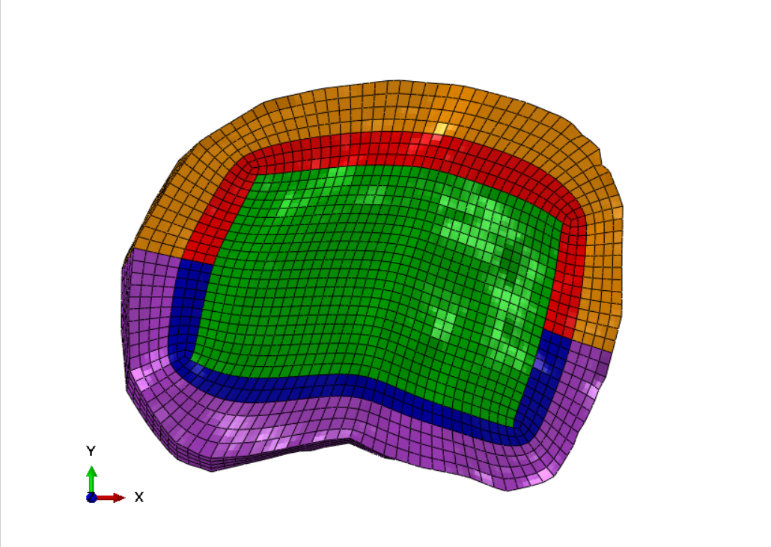


**Figure S3.** In GOH-1 model, different material parameters were adopted for different regions of the anulus, here shown: external ventral (orange), internal ventral (red), external dorsal (violet), internal dorsal (blue).

**Table S3.** Details of the constitutive laws parameters employed to model the nucleus pulposus.

| **Incompressible elastic** | $E=1 [MPa],\nu=0.499999995$ | [37] |
| --- | --- | --- |
| **Mooney-Rivlin-1** | $C_{10}=0.12 [MPa],$  $C_{01}=0.03 [MPa],$  $D=0.00667[MPa^{-1}]$ | [36] |
| **Mooney-Rivlin-2** | $C_{10}=0.12 [MPa],$  $C_{01}=0.03 [MPa],$  $D=0.667 [MPa^{-1}]$ | [38] |
| **Mooney-Rivlin-3** | $C_{10}=0.221 [MPa],$  $C_{01}=0.055 [MPa],$  $D=0.723\left[ MPa^{-1} \right]$ | [38] |
